# Supplementary figures and images for: Expression profiles of circular RNAs and interaction networks of competing endogenous RNAs in neurogenic bladder of rats following suprasacral spinal cord injury
Source: PeerJ. 2023 Sep 18;11:e16042. doi: 10.7717/peerj.16042 (PMC10512963; doi:10.7717/peerj.16042)

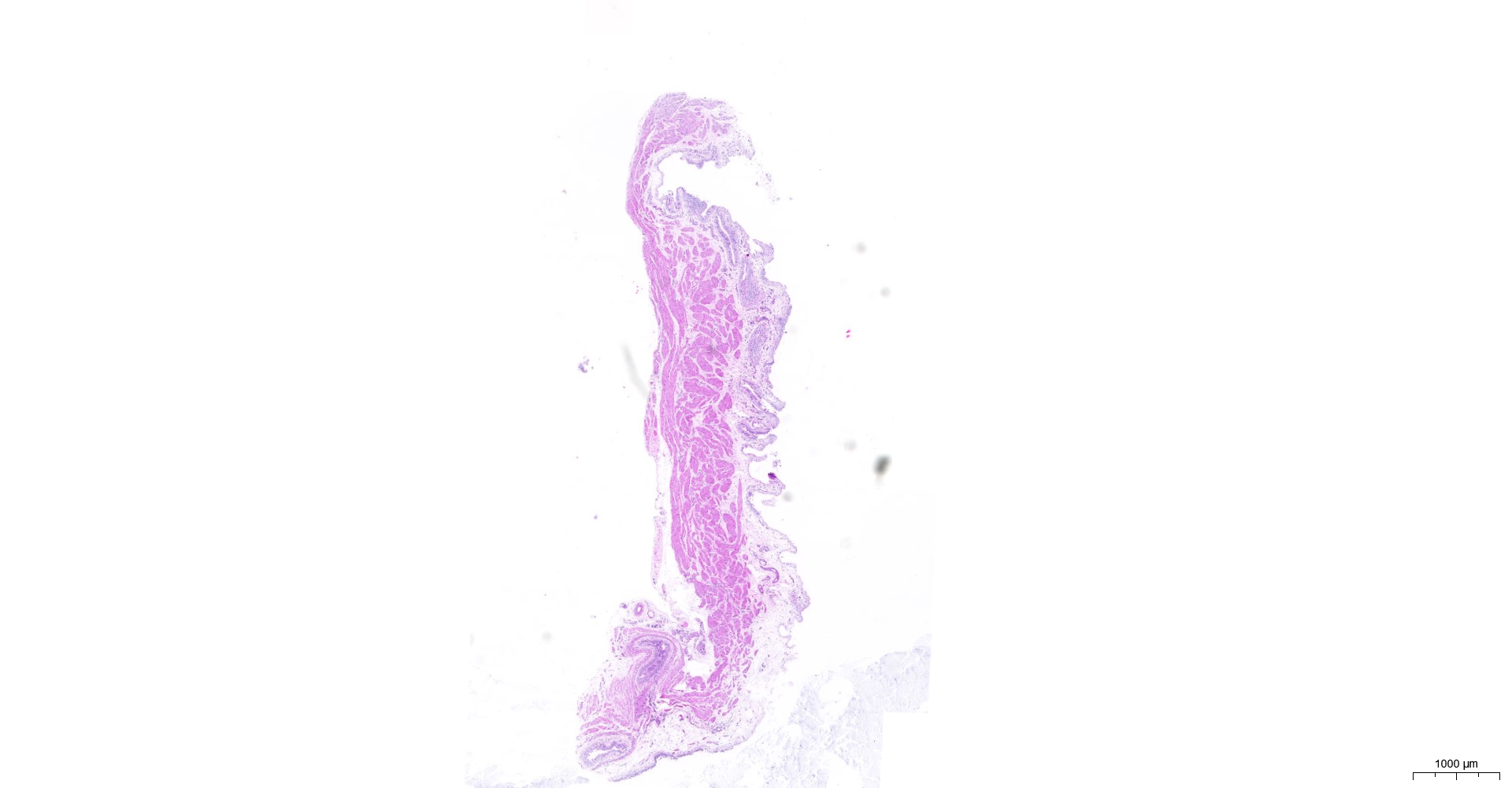

Supplement: Supplemental Information 3 [file peerj-11-16042-s003.jpg]

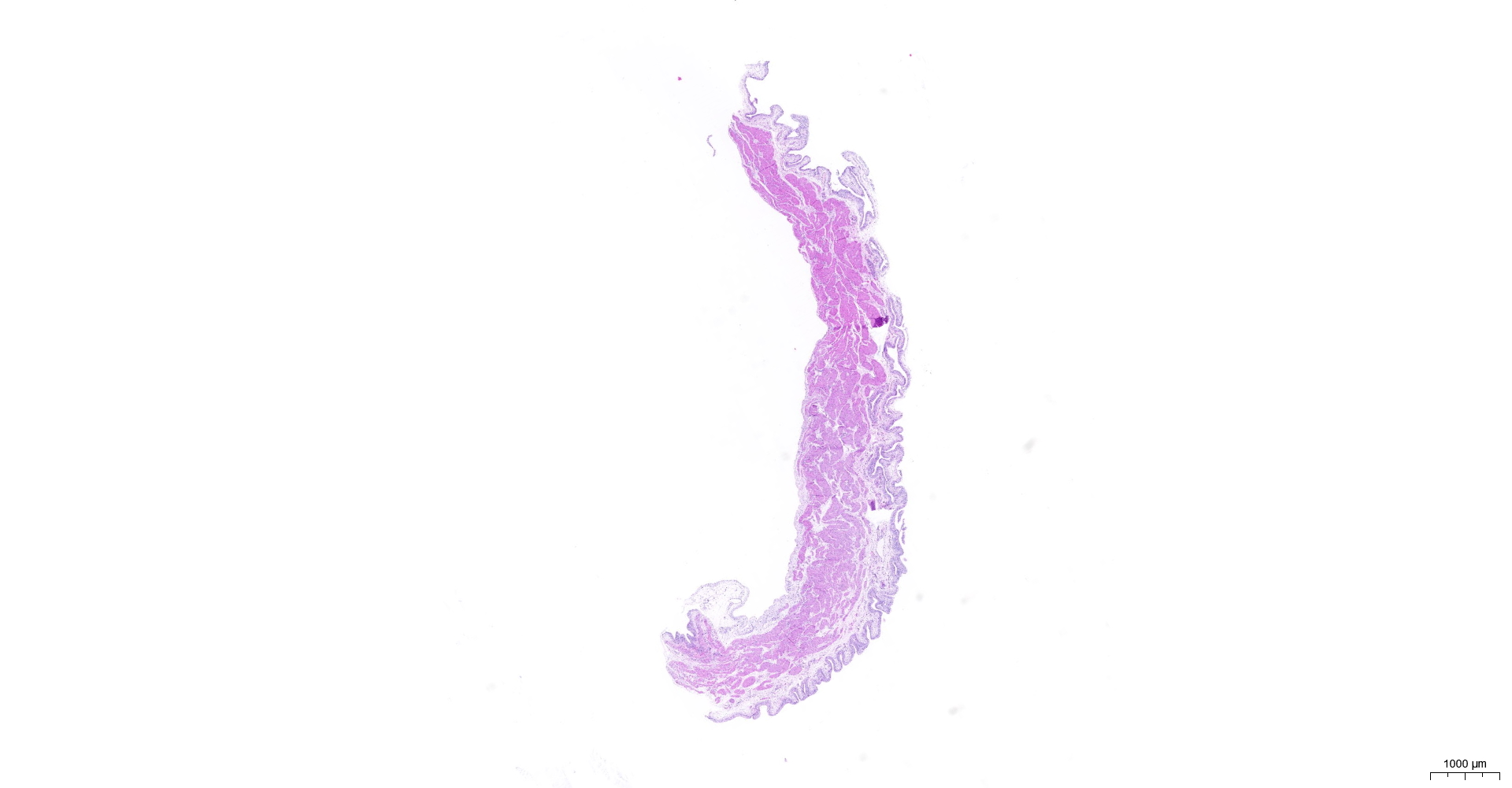

Supplement: Supplemental Information 4 [file peerj-11-16042-s004.jpg]

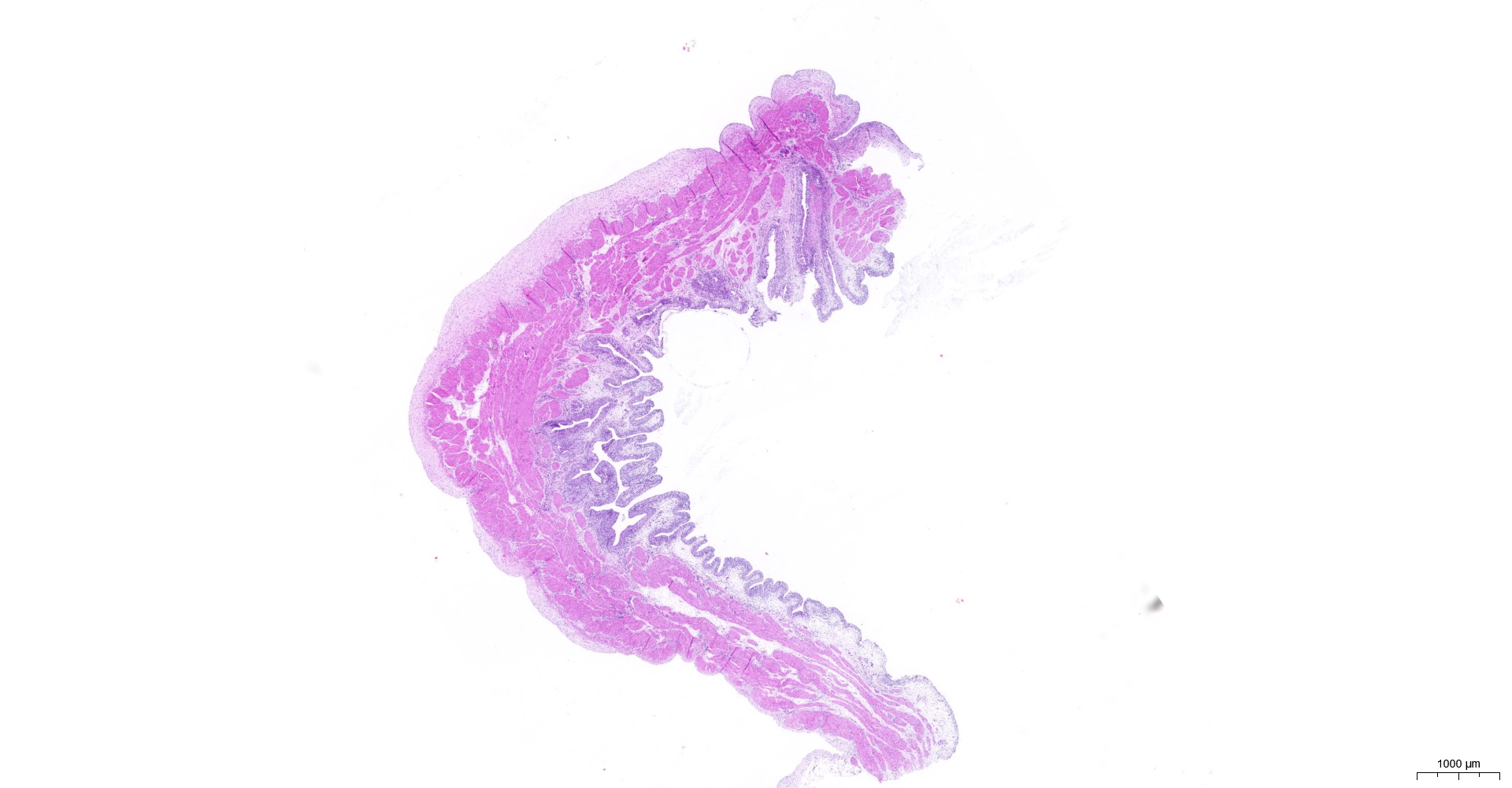

Supplement: Supplemental Information 5 [file peerj-11-16042-s005.jpg]

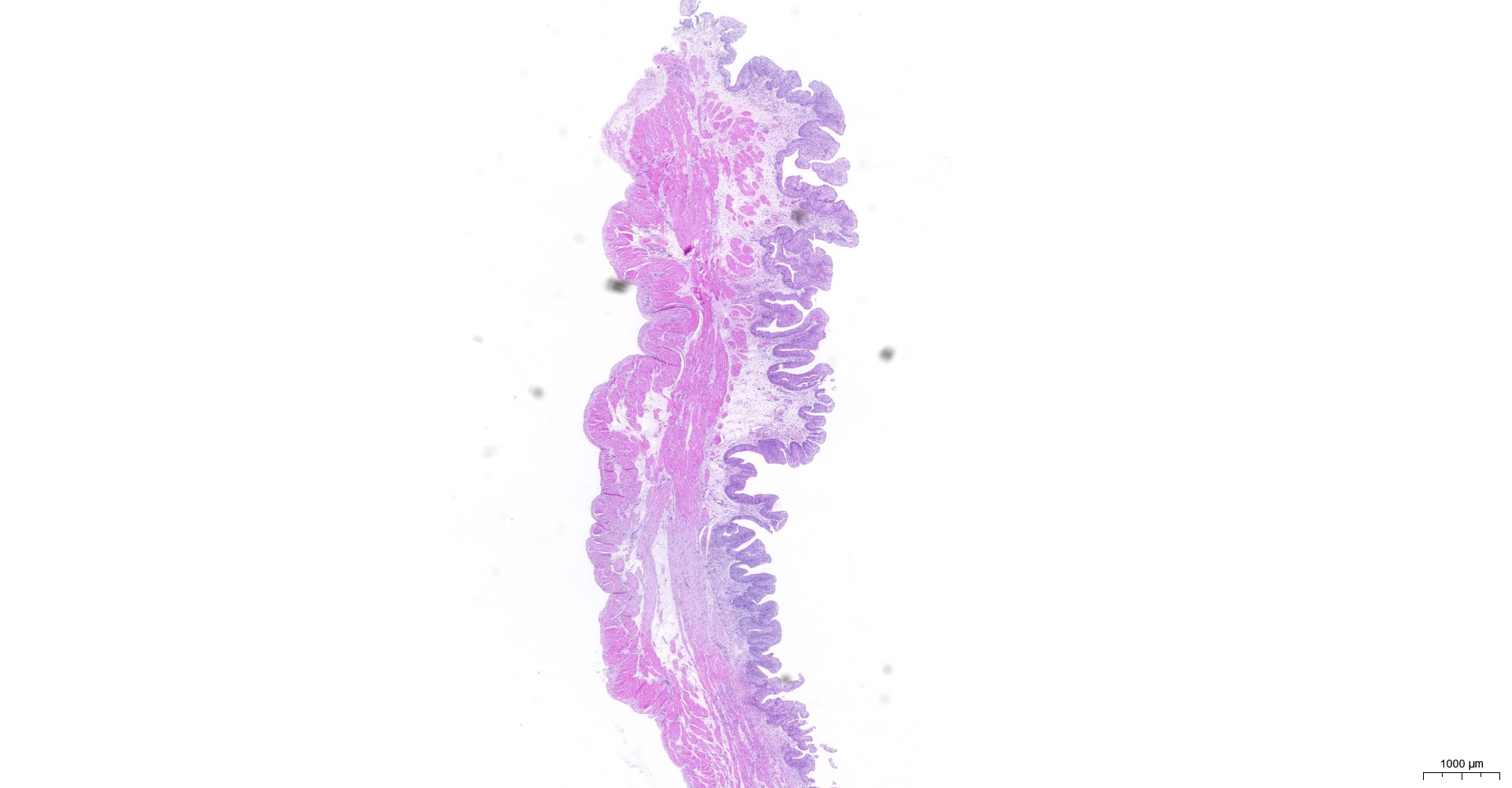

Supplement: Supplemental Information 6 [file peerj-11-16042-s006.jpg]

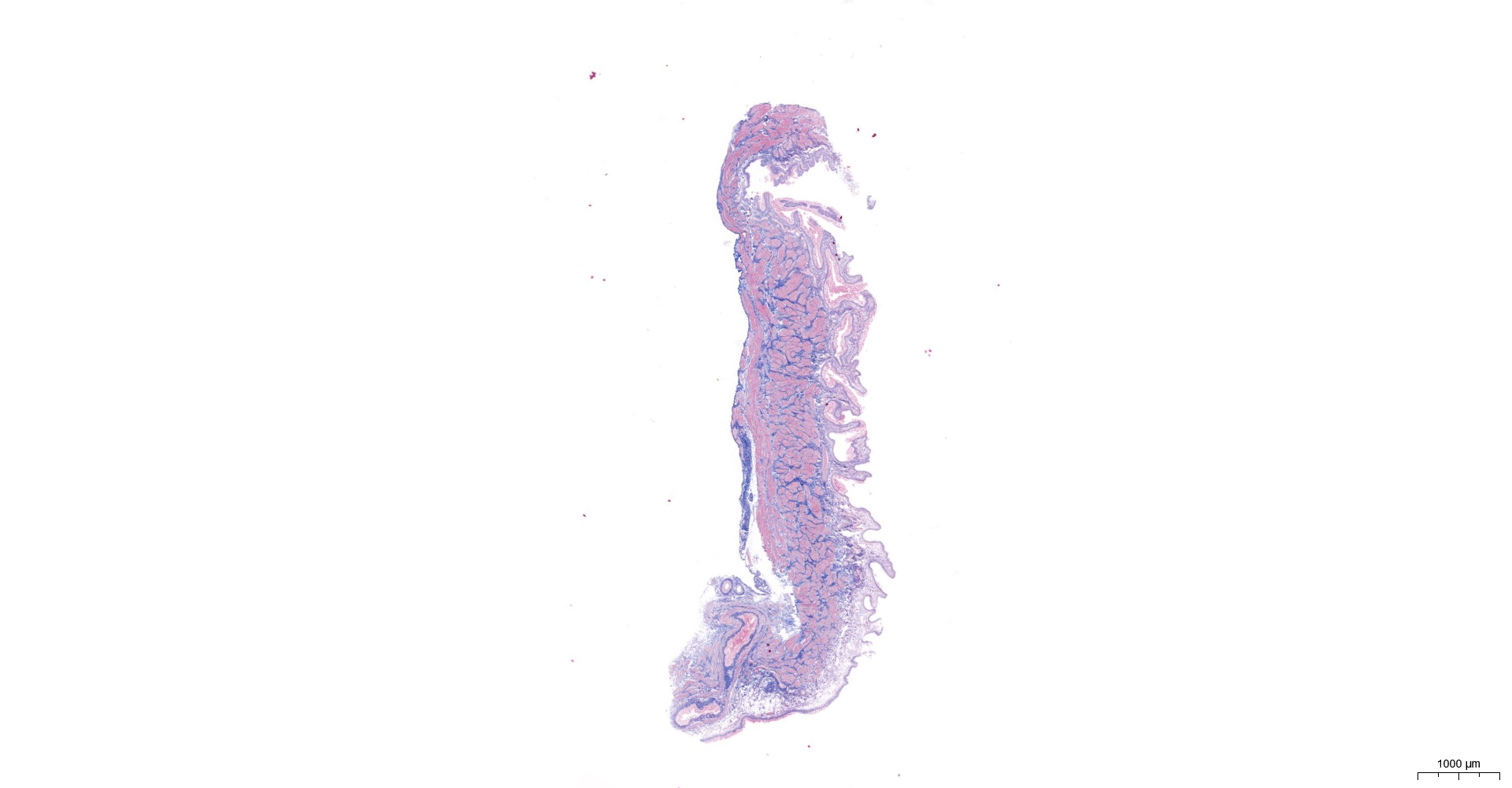

Supplement: Supplemental Information 7 [file peerj-11-16042-s007.jpg]

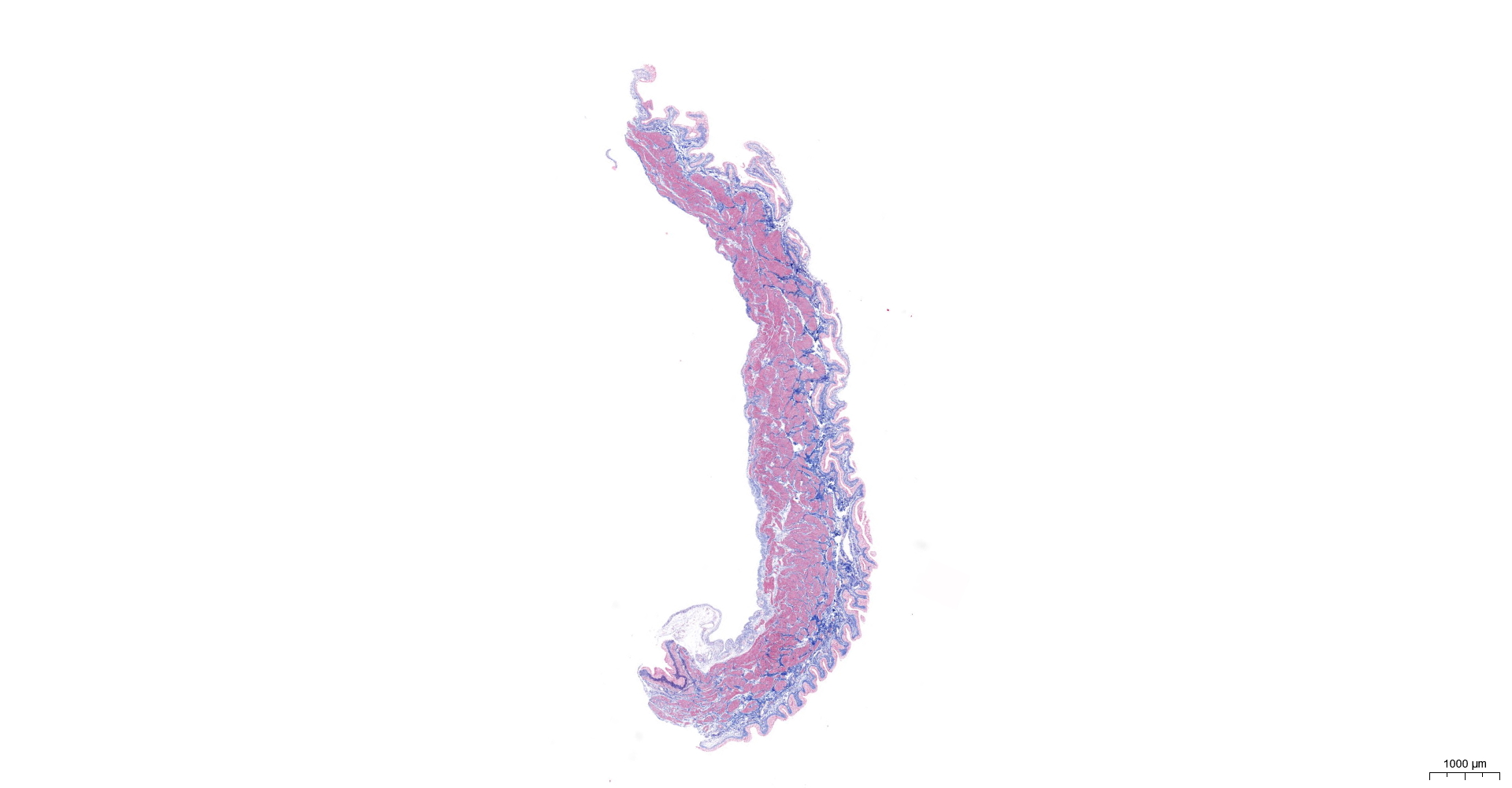

Supplement: Supplemental Information 8 [file peerj-11-16042-s008.jpg]

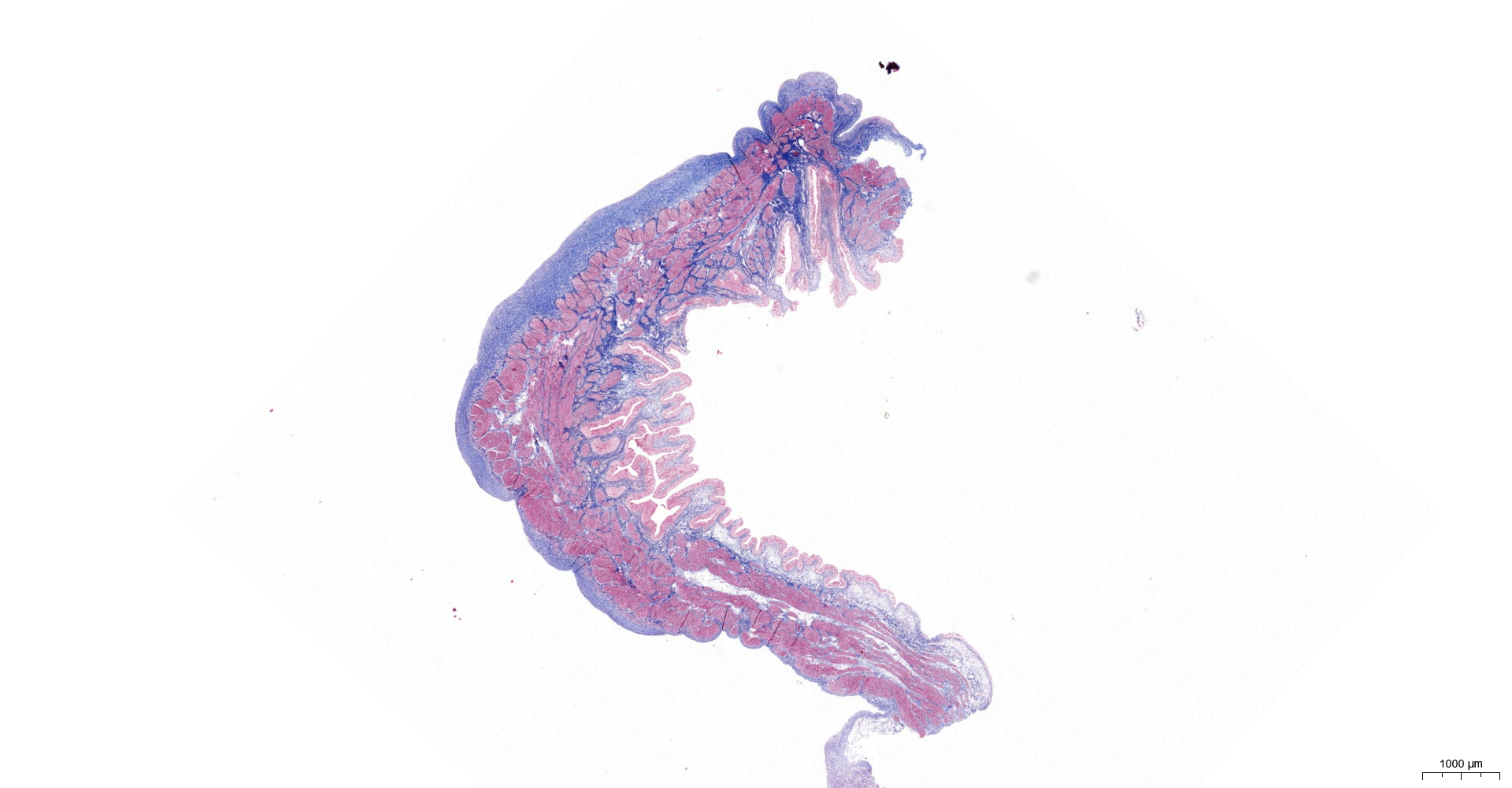

Supplement: Supplemental Information 9 [file peerj-11-16042-s009.jpg]

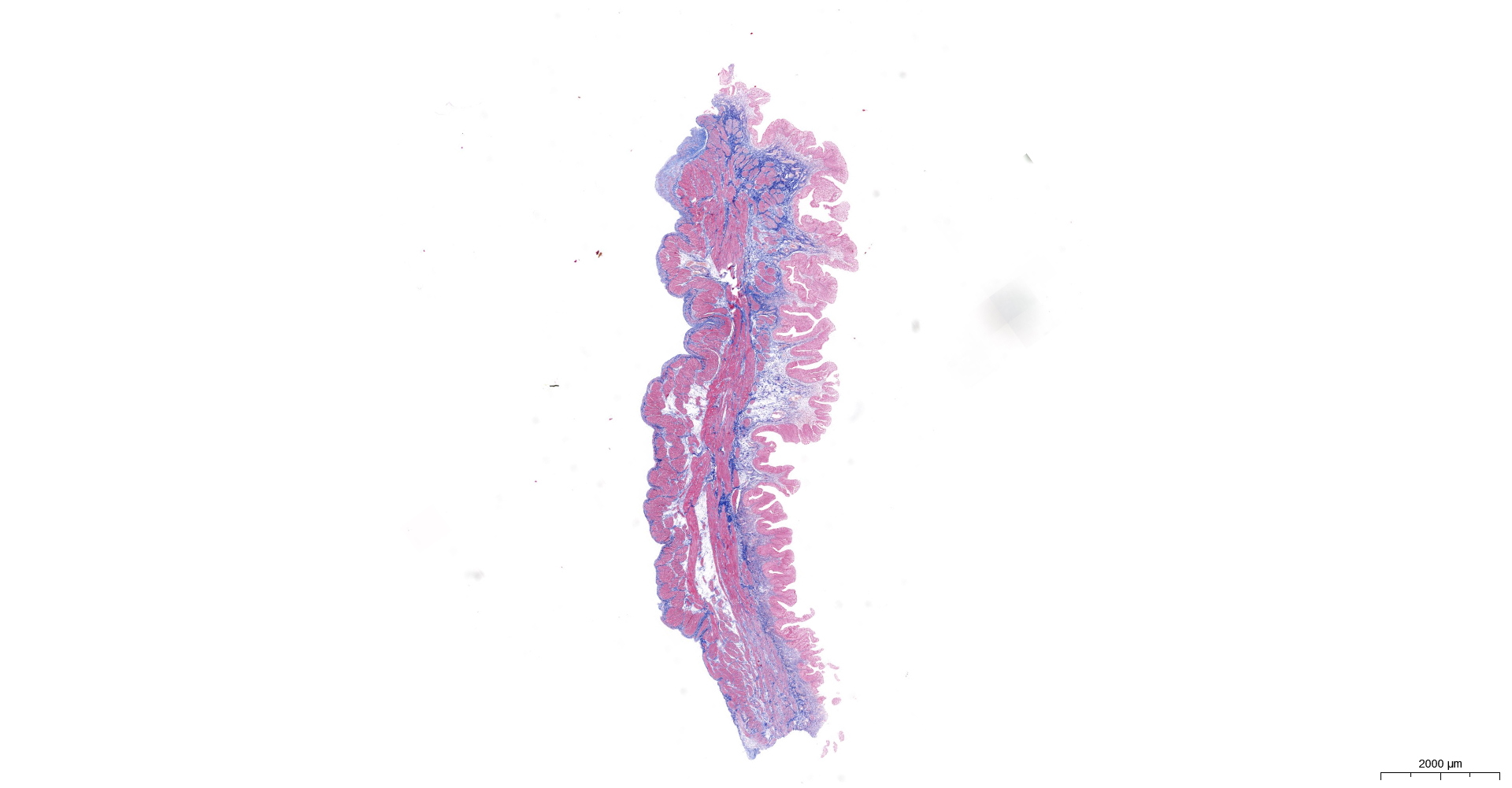

Supplement: Supplemental Information 10 [file peerj-11-16042-s010.jpg]
